# Navigating Chronic Pain in Patients with Cirrhosis: Unique Analgesic Considerations and Non-Pharmacologic Options

**DOI:** 10.1007/s11901-026-00735-9

**Published:** 2026-05-02

**Authors:** Lisa X. Deng, Jessica B. Rubin

**Affiliations:** 1 San Francisco Veterans Affairs Health Care System, San Francisco, California USA; 2 Division of Gastroenterology and Hepatology, Department of Medicine, University of California, San Francisco, San Francisco, California USA

**Keywords:** Chronic pain, Cirrhosis, Analgesics, Non-pharmacologic therapy, Opioids, Liver disease

## Abstract

**Purpose of Review:**

Chronic pain affects 40–80% of patients with chronic liver disease, yet management remains challenging due to impaired drug metabolism, limited evidence, and the absence of disease-specific guidelines. This review summarizes the evidence for pharmacologic and non-pharmacologic pain management strategies in patients with cirrhosis.

**Recent Findings:**

Nearly every analgesic class carries risk in patients with cirrhosis, and most prescribing recommendations are based on pharmacokinetic data and expert opinion rather than clinical trials. Opioid prescribing has declined in recent years, but nonopioid alternatives have not increased proportionally. Non-pharmacologic modalities have been scarcely studied in this population. Shared comorbidities and risk factors, including alcohol use, metabolic disease, sleep disturbances, and psychiatric conditions, represent modifiable therapeutic targets that may improve pain while altering liver disease trajectory.

**Summary:**

Pain management in patients with cirrhosis requires an individualized, multimodal approach integrating pharmacologic therapy, non-pharmacologic interventions, and comorbidity management. Further research, including both well-designed observational studies and prospective trials, is needed to generate evidence-based guidance for this underserved population.

## Introduction

Chronic pain affects 40–80% of patients with chronic liver disease and is associated with decreased quality of life, increased disability, and high rates of health care utilization [1–3]. Yet, pain in this population is often undertreated [2]. Pain in patients with chronic liver disease is complex and incompletely understood, intersecting with metabolic comorbidities, substance use disorders, psychiatric disease, and social determinants of health [1, 4]. Management is further complicated by the convergence of pharmacologic, patient, disease, clinician and system-level factors that together make this one of the most frequently encountered and challenging clinical problems in hepatology.

Hepatic impairment alters drug metabolism across nearly every analgesic class, increasing the risk of adverse effects such as renal injury, hepatic encephalopathy, and gastrointestinal (GI) bleeding [5]. Additionally, patients with advanced liver disease are routinely excluded from analgesic trials, and as a result, most prescribing recommendations are based on pharmacokinetic data and expert opinion rather than direct clinical evidence. Patients are often counseled to avoid commonly used over-the-counter analgesics, including acetaminophen and NSAIDs, while alternatives such as opioids and adjuvant analgesics (e.g., gabapentin, antidepressants) may carry their own risks in this population. No guidelines currently exist for the management of chronic pain in patients with chronic liver disease or cirrhosis, and nonpharmacologic modalities have been scarcely investigated in this population.

This review synthesizes the available evidence on the safety and efficacy of pharmacologic analgesics in advanced liver disease and examines emerging data on non-pharmacologic interventions. We also discuss how management of comorbidities and risk factors that are shared between cirrhosis and chronic pain may itself serve as a pain management strategy. Given the limitations of any single therapeutic approach, we advocate for an individualized, multimodal framework that integrates pharmacologic, non-pharmacologic, and comorbidity-directed strategies.

## Pharmacotherapy

While data on analgesic safety and efficacy in patients with cirrhosis remain limited, a general principle of cautious initiation at low doses with slow titration applies across drug classes (Table 1). The following sections summarize the available evidence for individual analgesic classes, beginning with opioids, which remain the most commonly prescribed and most debated agents in this population.

**Table 1:** Pharmacologic Analgesic Options in Patients with Cirrhosis

Drug Class	Key Considerations in Liver Disease	Evidence Base	Dosing in Patients with Cirrhosis
**Opioids** *Preferred: fentanyl, oxycodone, hydromorphone* *Avoid: tramadol, codeine, morphine, methadone* *Data lacking: buprenorphine*	Impaired metabolism and increased bioavailability Associated with hepatic encephalopathy, increased health care utilization, worse post-transplant outcomes *Note:* Tramadol is often perceived as safer but has unpredictable pharmacokinetics in cirrhosis and may cause hypoglycemia and hyponatremia	Observational studies, pharmacokinetic studies	Low dose, extended intervals
**Acetaminophen**	Generally tolerated among patients with chronic liver disease Caution with active alcohol use (glutathione depletion, CYP P450 induction), acute viral hepatitis, critical illness	Small RCTs, observational studies; no long-term prospective data	Up to 2 g/day thought to be safe
**NSAIDs** *Non-selective: ibuprofen, naproxen* *COX-2 selective: celecoxib* *Topical NSAIDs*	Increased risk for renal injury, blunting of diuretic response, GI bleeding COX-2 inhibitor safety profile remains unknown Topical NSAIDs have minimal systemic exposure	Case–control and case-crossover studies; no prospective data	Generally avoid oral NSAIDs in patients with cirrhosis
**Gabapentinoids** *Gabapentin, pregabalin*	No hepatic metabolism; renally excreted Associated with dizziness, sedation, fall risk Possible association with increased HE risk	Small RCT (terminated early), observational data; very limited overall	Start low, titrate slowly. Renal dosing required
**Antidepressants** *TCAs; SNRIs (venlafaxine, duloxetine); SSRIs*	TCAs: possible accumulation, anticholinergic effects may worsen HE, possible benefit on liver fibrosis SNRIs: increased bioavailability; duloxetine associated with liver injury SSRIs: less efficacious for neuropathic pain; increased GI bleeding risk	Minimal cirrhosis-specific studies, only observational	Start low, titrate slowly. Individualize based on comorbidity profile, cirrhosis complications, and pain type
**Ketamine**	Associated with liver injury and cholangiopathy	No cirrhosis-specific studies	Generally avoid in patients with cirrhosis
**Topical Analgesics** *Lidocaine patches, capsaicin, topical NSAIDs*	Minimal systemic absorption May reduce need for oral analgesics Low rates of systemic adverse events; local reactions may occur	No cirrhosis-specific studies	Standard dosing; may serve as early treatment option given low risk profile
**Analgesic Injections** *Intra-articular, nerve blocks, intrathecal*	Peripheral joint injections generally low bleeding risk even in anticoagulated patients Nerve block risk variable; intrathecal slightly higher bleeding risk but overall still low	No cirrhosis-specific studies	Best if neuropathology identified Assess bleeding risk

*COX-2* cyclooxygenase-2, *CYP* cytochrome P450, *GI* gastrointestinal, *HE* hepatic encephalopathy, *NSAIDs* nonsteroidal anti-inflammatory drugs, *RCT* randomized controlled trial, *SNRIs* serotonin-norepinephrine reuptake inhibitors, *SSRIs* selective serotonin reuptake inhibitors, *TCAs* tricyclic antidepressants

### Opioids

Opioids are widely prescribed to patients with cirrhosis, with reported prevalence ranging from 25 to 77% depending on the population and how use is defined [6–9]. Reduced hepatic enzyme capacity and portosystemic shunting increase opioid bioavailability in patients with advanced liver disease. Hypoalbuminemia, impaired biliary excretion, renal dysfunction, and sarcopenia further predispose to drug accumulation [10]. Although opioids predominantly undergo hepatic metabolism, specific metabolic pathways vary by agent and pharmacokinetic data in patients with severe hepatic impairment are limited.

A key concern in cirrhosis is the potential for opioids to precipitate or exacerbate hepatic encephalopathy (HE). In animal models, upregulation of central μ-opioid receptors as a response to decreased endogenous opioids leads to increased sensitivity to the neuroinhibitory effects of exogenous opioids [11]. Blood–brain barrier alterations and opioid-mediated effects on the gut–brain axis, including changes in intestinal permeability, gut dysbiosis, and opioid-induced constipation, may further contribute to HE risk [12]. Some observational studies have found an increased risk of HE among patients with cirrhosis using opioids [13, 14], whereas others have not [12, 15]. The true real-world effect of opioids on HE risk in this population remains unknown.

There are no clinical trials comparing different opioid agents in patients with cirrhosis, and selection is guided largely by expert opinion based on pharmacokinetic profiles. Fentanyl has the most favorable metabolic profile, but is less practical for the outpatient setting, as the lowest available transdermal patch dose may still be too high for patients with significant hepatic impairment [16]. Expert guidance generally recommends low-dose hydromorphone or oxycodone with extended dosing intervals as first-line agents [17]. Tramadol is often perceived as a “safer” opioid alternative; however, it requires hepatic transformation to its active metabolite via CYP2D6, resulting in unpredictable pharmacokinetics in patients with liver disease. In the general population, tramadol also carries risks for hypoglycemia, hyponatremia, and serotonin syndrome, and a recent systematic review found only modest pain relief with increased serious adverse events [18–20].These risks are particularly concerning in patients with cirrhosis, who are already predisposed to metabolic derangements. Despite these concerns, tramadol use has been shown to be more common among inpatients with cirrhosis compared to those without, particularly in patients with decompensated disease [21]. Codeine should similarly be avoided due to its dependence on hepatic transformation to active metabolites [10]. Morphine is less preferred due to extensive first-pass hepatic metabolism, resulting in increased bioavailability and decreased clearance in hepatic impairment [22]. Methadone has a long and variable half-life with multiple drug interactions.

Buprenorphine, a partial μ-opioid receptor agonist, is increasingly prescribed for both chronic pain and opioid use disorder. Early studies raised concern for transaminase elevations in patients with chronic liver disease [23], although subsequent small studies have not found an association [24, 25]. These studies largely excluded patients with advanced liver disease. Given the higher prevalence of opioid use disorder and chronic pain among patients with cirrhosis, more data are needed on the safety, efficacy, and dosing of buprenorphine in this population for both indications.

Despite theoretical concerns based on impaired metabolism, few studies have directly compared rates of opioid-related adverse events between patients with and without cirrhosis; those that have suggest similar rates [21]. Several observational studies have found associations between opioid use and increased health care utilization among patients with cirrhosis [12, 26, 27], and worse outcomes among the subset that undergo liver transplant [28]. Beyond these findings, clinical outcomes data in this population are limited, underscoring the gap between pharmacokinetic concerns and available evidence. In the context of national efforts to reduce opioid prescribing, opioid use among patients with cirrhosis has declined in recent years, increasing the importance of understanding the risks and benefits of nonopioid alternatives in this population [9].

## Nonopioid Analgesics

As opioid prescribing has declined, understanding the safety and efficacy of nonopioid alternatives in patients with cirrhosis has become increasingly important. However, data for most nonopioid classes in this population are similarly limited.

### Acetaminophen

The well-known association between acetaminophen overdose and acute liver failure has led to the perception among clinicians that acetaminophen is not safe in patients with chronic liver disease [29]. However, small studies support the safety of acetaminophen in patients with cirrhosis. Although chronic liver disease raises theoretical concern for increased toxicity, pharmacokinetic studies in patients with cirrhosis demonstrate reduced drug clearance without increased formation of toxic metabolites [30–32]. Small studies, including a RCT and two case–control studies, suggest that short-term acetaminophen use at doses up to 2–4 g/day is tolerated in patients with chronic liver disease [33–35].

Caution should be exerted in certain populations. Chronic alcohol use has been associated with hepatotoxicity from acetaminophen, likely due to both glutathione depletion and increased cytochrome P450 activity, which may raise levels of toxic metabolites [36]. Short-term acetaminophen use appears to be tolerated in this population [37, 38]. A retrospective cohort study found that in critically ill patients with cirrhosis, early acetaminophen use, even at low doses, was associated with increased mortality [39]. Prior literature also shows that patients with acute viral hepatitis exposed to acetaminophen have increased risk for adverse outcomes [40, 41]. In clinical practice, up to 2 g/day is generally recommended for patients with cirrhosis, though prospective long-term safety data are lacking.

### Nonsteroidal Anti-Inflammatory Drugs (NSAIDs)

NSAIDs are commonly used for musculoskeletal pain, and surveys suggest that non-hepatologists may be more likely to recommend NSAIDs over acetaminophen in patients with cirrhosis [29]. However, in patients with cirrhosis, impaired hepatic function alters drug metabolism and increases bioavailability for most types of NSAIDs. By inhibiting prostaglandin synthesis, NSAIDs reduce compensatory renal vasodilation, thereby impairing renal perfusion, decreasing glomerular filtration rate, and promoting sodium retention, which can precipitate or worsen ascites [42]. In patients with cirrhosis and ascites, NSAIDS have been shown to blunt the natriuretic response to diuretics [42]. Several small studies have also shown that NSAIDs are associated with acute kidney injury in patients with cirrhosis [43, 44].

NSAIDs have a well-known association with gastrointestinal bleeding [45]. A case–control study found that cirrhotic patients using NSAIDs had nearly three-fold increased odds of portal hypertension-related bleeding [46].Additionally, a case-crossover study demonstrated elevated risk with non-selective NSAIDs but not with celecoxib, though the study was likely underpowered to detect an effect [47].

Because COX-2 selective inhibitors spare COX-1-mediated platelet aggregation and gastric mucosal protection, they may carry a lower bleeding risk than non-selective agents. However, COX-2 is also expressed in the kidney, and whether COX-2 inhibitors truly offer a safer overall profile in patients with cirrhosis remains an open question. Topical NSAIDs likely confer minimal systemic exposure; however, cirrhosis-specific safety data are lacking.

### Gabapentinoids

Gabapentin and pregabalin undergo no hepatic metabolism and are renally excreted, making them appealing therapeutic options for patients with hepatic impairment [48]. In part for this reason, gabapentinoid prescribing in the general population and among patients with cirrhosis has increased substantially in recent years [9, 49, 50]. However, there is little data on drug safety in this population. Gabapentinoids are associated with dose-dependent dizziness and sedation, which can increase fall risk [51]. These concerns are recognized in other high risk populations, such as older adults, and are equally relevant in patients with cirrhosis, particularly those with HE [52]. Observational data suggest that gabapentinoid use in patients with cirrhosis may be associated with increased HE risk, though confounding by indication is difficult to exclude [14]. One RCT evaluated pregabalin for treatment of muscle cramps in patients with cirrhosis but was terminated early due to insufficient recruitment; among those enrolled, no differences in adverse events were observed between pregabalin and placebo [53]. Another small study evaluating pregabalin for restless leg syndrome in patients with cirrhosis found that treatment discontinuation commonly occurred at higher doses due to adverse effects [54]. These agents should be started at low doses and titrated slowly in patients with advanced liver disease, with close attention to renal function and cognitive status.

### Antidepressants

Antidepressants are a cornerstone of chronic pain management, particularly given the strong bidirectional relationship between pain and psychiatric comorbidities, as discussed further below. However, there is little data on their safety and efficacy in patients with advanced liver disease. Tricyclic antidepressants (TCAs) primarily undergo hepatic metabolism, leading to possible accumulation, although pharmacokinetic data in patients with cirrhosis are lacking. Anticholinergic effects may be problematic in this population, as constipation can increase the risk of HE. Interestingly, there is emerging evidence that TCAs may improve liver fibrosis and reduce portal hypertensive complications, a potential dual benefit that warrants further investigation [55, 56].

Serotonin-norepinephrine reuptake inhibitors (SNRIs), such as venlafaxine and duloxetine, have increased bioavailability and decreased clearance in patients with hepatic impairment [57]. Duloxetine has been associated with liver injury, and the manufacturer advises against prescribing it in patients with substantial alcohol use or chronic liver disease [58, 59]. SSRIs may be less efficacious than TCAs for neuropathic pain specifically, but may have a role when depression or anxiety is a significant contributor to the pain experience. However, SSRIs carry concern for increased GI bleeding, which requires careful consideration in patients with cirrhosis and portal hypertension [60, 61]. Given the limited evidence, selection among antidepressant classes in this population should be individualized based on the patient’s comorbidity profile, pain type, and risk factors for adverse effects.

### Topical Analgesics

Topical analgesics including lidocaine patches and creams, capsaicin, and topical NSAIDs, have not been studied in patients with cirrhosis, but are generally considered safe given minimal systemic absorption [62, 63]. Local skin reactions can occur, but systemic adverse events are rare. In other chronic pain populations, topical agents have been shown to reduce the need for oral analgesics, supporting their role as an early treatment strategy in patients with cirrhosis for whom systemic agents carry greater risk [64].

### Ketamine

Patients are increasingly inquiring about ketamine for chronic pain, often in the context of ketamine clinics or intranasal protocols used for comorbid depression. Despite this growing interest, the evidence for chronic pain is weak outside of complex regional pain syndrome [65, 66]. Ketamine is an N-methyl-D-aspartate (NMDA) antagonist that undergoes extensive hepatic metabolism. While studies of ketamine have excluded patients with advanced liver disease, ketamine has been associated with liver injury, and cases of cholangiopathy and fibrosis have been observed with chronic use [67–69]. Beyond hepatotoxicity, ketamine’s dissociative and cognitive effects are of particular concern in a population already at risk for HE. Thus, ketamine should generally be avoided among patients with cirrhosis. Yet in patients with refractory chronic pain with limited other options, ketamine may be considered on an individual basis weighing the risks and benefits.

### Analgesic Injections

Interventional therapies, including interarticular injections, nerve blocks, and intrathecal injections, have also not been evaluated in patients with cirrhosis. In chronic pain populations, these therapies can be effective components of a multimodal strategy, but are generally most appropriate for patients with identifiable neuropathology rather than centrally mediated pain [70]. In patients with cirrhosis, bleeding risk must be carefully considered given the prevalence of thrombocytopenia and coagulopathy in this population. Peripheral joint injections carry relatively low bleeding risk; substantial data from patients on anticoagulation support the safety of intra-articular injections in patients on antithrombotic therapy [71, 72]. Risk with peripheral nerve blocks varies depending on the targeted nerve [73]. Intrathecal injections carry the highest risk due to potential for epidural hematoma, though absolute incidence remains low even in anticoagulated patients [74].

In practice, concern about bleeding in patients with cirrhosis may lead interventionalists to avoid these procedures altogether, potentially limiting access to an effective treatment modality. Better characterization of procedural bleeding risk in this population, along with improved communication between hepatologists and pain specialists, could help guide shared decision-making and expand the use of these therapies where appropriate.

## Non-Pharmacologic Options

Given the significant limitations of pharmacotherapy in patients with cirrhosis, non-pharmacologic approaches are not merely complementary but may need to serve as a foundation of pain management in this population. However, whereas the pharmacotherapy literature in cirrhosis has focused primarily on safety, the challenge with non-pharmacologic modalities is the near-absence of efficacy data specific to this population. Most evidence is extrapolated from the broader chronic pain literature, where several modalities have demonstrated benefit (Table 2).

**Table 2:** Non-pharmacologic Interventions, Supplements, and Cannabis for Pain in Patients with Cirrhosis

Modality	Evidence in Chronic Pain	Evidence in Cirrhosis	Considerations in Cirrhosis
*NON-PHARMACOLOGIC APPROACHES*			
Cognitive behavioral therapy and self-management programs	Small to moderate improvements in pain intensity; strong evidence base across multiple chronic pain conditions	LEAP program described (12-week phone-based intervention); no clinical outcomes reported. Can be delivered virtually	Psychiatric comorbidities, sleep disturbances
Mindfulness-based interventions	Small to moderate effect on pain intensity	Not studied in cirrhosis	Psychiatric comorbidities, sleep disturbances
Exercise/Physical therapy	Beneficial for chronic pain; heterogeneous data across modalities	Small studies show improvements in physical function and quality of life (not pain-specific)	Obesity, frailty, sarcopenia. Modify for ascites, fatigue, deconditioning
Acupuncture/TEAS	Moderate evidence for small improvements in pain	One RCT of TEAS in HCC patients (~ 40% Child–Pugh B) showed significant improvements in pain and QOL	Theoretical bleeding risk with needles in coagulopathy/thrombocytopenia (likely low); needle-free modalities (TEAS) available
*SUPPLEMENTS FOR MUSCLE CRAMPS*			
Pickle juice	Observed benefit for muscle cramps in general population	One small RCT showed significant cramp reduction; no sodium-related complications observed	Monitor fluid retention in patients with ascites
Taurine	None	Small RCT showing improvement in cramp frequency, duration, and severity at 2 g/day; no adverse effects observed	
Branched chain amino acids (BCAAs)	None	Small RCT showing improvement in cramp frequency	May also improve hepatic encephalopathy. Adverse effects: nausea, diarrhea
L-carnitine	None	Very limited; small single-arm studies and retrospective data suggest possible benefit. Meta-analysis did not confirm benefit for HE	
*SUPPLEMENTS FOR CHRONIC PAIN*			
Omega-3 fatty acids	Systematic reviews support benefit for inflammatory and rheumatoid arthritis pain	Not studied for pain in cirrhosis	Generally safe
Curcumin	Small trials suggest efficacy comparable to NSAIDs for osteoarthritis	Not studied for pain in cirrhosis	Turmeric-based supplements increasingly implicated in drug-induced liver injury; use with caution
Vitamin D	Associated with improvement in chronic widespread pain; inconsistent relationship between serum levels and outcomes	Not studied for pain in cirrhosis	Deficiency common in cirrhosis; supplementation often indicated regardless
Acetyl-L-carnitine	Benefit for neuropathic pain, particularly diabetic neuropathy, in small RCTs	Not studied for pain in cirrhosis	
*CANNABIS*			
Cannabis	Small benefit in chronic pain populations	No pain-specific data. Observational data suggest potentially favorable liver-related outcomes, but one study reported association with increased ascites	Increasing prevalence of use; unique pharmacologic considerations; important area for future study

*BCAAs* branched-chain amino acids, *HCC* hepatocellular carcinoma, *LEAP* Liver Education About Pain, *MASLD* metabolic dysfunction-associated steatotic liver disease, *NSAIDs* nonsteroidal anti-inflammatory drugs, *QOL* quality of life, *RCT* randomized controlled trial, *TEAS* transcutaneous electrical acupoint stimulation

### Behavioral and Psychological Interventions

Cognitive behavioral therapy (CBT) and mindfulness-based interventions have the strongest evidence base in chronic pain populations, with data showing small to moderate improvements in pain intensity [75–78]. These approaches may be particularly relevant for patients with cirrhosis, in whom psychiatric comorbidities including depression and anxiety are prevalent and contribute to the pain experience [1]. One study in patients with cirrhosis described the development of a pain self-management program, Liver Education About Pain (LEAP), a 12-week modular intervention delivered by phone via individual and group sessions with a health coach [79]. This study described the intervention design process but clinical outcomes have not yet been reported. An advantage of behavioral interventions is that they can be delivered virtually, which may help address access barriers common in this medically complex population [75]. Further trials are needed to evaluate the efficacy of these approaches for pain in patients with cirrhosis.

### Exercise and Movement-Based Therapies

Exercise and physical therapy interventions have demonstrated benefit for chronic pain in the general population, though data are heterogenous. These interventions have not been studied for pain specifically in patients with cirrhosis, but small studies of exercise based interventions for patients with cirrhosis have demonstrated improvements in physical function and quality of life [80, 81]. Yoga has shown benefit in chronic pain populations and is currently being studied as an intervention for metabolic dysfunction-associated steatotic liver disease (MASLD) with encouraging results in small RCTs [82–85]. In patients with MASLD-related cirrhosis, exercise may be particularly important, as weight loss and physical activity have the potential to slow or reverse hepatic fibrosis, offering benefits beyond pain management alone. Exercise-based approaches have the added advantage of addressing sarcopenia and frailty, which are prevalent in cirrhosis and may themselves contribute to chronic pain, as discussed further below.

### Acupuncture

Acupuncture has demonstrated efficacy for chronic pain, particularly back pain, in the general population, but limited data exist in patients with advanced liver disease [86, 87]. There is theoretical bleeding risk in patients with thrombocytopenia and coagulopathy, though this risk is likely low; acupuncture has not been associated with major bleeding events in patients on anticoagulation [88]. Needle-free alternatives also exist, such as transcutaneous electrical acupoint stimulation (TEAS), which delivers electrical stimulation to acupuncture points through surface electrodes. In one RCT, patients with hepatocellular carcinoma, approximately 40% of whom had Child–Pugh class B cirrhosis, who received TEAS demonstrated significant improvements in both pain and quality of life compared with controls [89]. This study represents one of the few prospective trials of any non-pharmacologic pain intervention in patients with advanced liver disease.

### Other Non-Pharmacologic Modalities

Several other non-pharmacologic modalities are used in chronic pain populations, including massage therapy, manual therapy (e.g. joint mobilization), chiropractic care, heat and cryotherapy, transcutaneous electrical nerve stimulation, and virtual reality therapy. Evidence for these treatments in the general population is low to moderate, and none have been studied in patients with cirrhosis. These approaches could nonetheless be considered on an individual basis given their generally favorable safety profiles and patient preference.

## Supplements and Cannabis

Patients with cirrhosis and chronic pain frequently use supplements and cannabis, often without clinical guidance. While these agents are pharmacologically active, they fall outside traditional analgesic classes and have distinct evidence bases that warrant separate consideration.

### Supplements

In the general population, several supplements have shown modest evidence for chronic pain, particularly inflammatory and musculoskeletal pain. Omega-3 fatty acids have the most consistent data, with systematic reviews supporting benefit in rheumatoid arthritis and other inflammatory conditions [90, 91]. Curcumin has demonstrated anti-inflammatory properties, with small trials suggesting efficacy comparable to NSAIDs for osteoarthritis [92, 93]. However, turmeric-based supplements have been increasingly implicated in drug-induced liver injury and thus, should be used with caution in patients with underlying liver disease. Vitamin D supplementation has been associated with improvement in chronic widespread pain, though the relationship between serum levels and pain outcomes is inconsistent [94]. Acetyl-L-carnitine has shown benefit for neuropathic pain, particularly diabetic neuropathy, in small RCTs [95]. None of these supplements have been evaluated for pain in patients with cirrhosis. Given their generally favorable safety profiles, they may warrant consideration on an individual basis, though potential interactions with liver disease and its treatments should be considered.

While data on supplements for chronic pain in cirrhosis are lacking, muscle cramps—which are extremely common in cirrhosis—represent one area where cirrhosis-specific evidence does exist. Pickle juice has been observed to help muscle cramps in the general population, possibly through a neural reflex triggered by oropharyngeal sensory receptors [96, 97]. One small RCT in patients with cirrhosis showed significant reduction in cramp severity with no sodium-related complications, even among those with ascites [98]. Several supplements have also been studied for muscle cramps in cirrhosis with limited data. Small RCTs suggest benefit for taurine (2 g/day) and branched-chain amino acids (BCAAs), with no significant adverse effects [99–103]. Data for L-carnitine are very limited [104, 105].

### Cannabis

Cannabis use for chronic pain is increasing, with studies showing small benefit in the general population [106]. In large observational studies, cannabis has been associated with potentially favorable liver-related outcomes, including lower rates of cirrhosis [107, 108], reduced prevalence of MASLD [109], and decreased hepatic decompensation and hospital admissions [110, 111]. However, a separate retrospective analysis reported an association between cannabis and increased ascites [112]. There is no data to date on the efficacy and safety of cannabis for the treatment of chronic pain in patients with cirrhosis. Given the increasing prevalence of cannabis use among patients with liver disease and the unique pharmacologic considerations in this population, this represents an important area for future study.

## Management of Comorbidities and Risk Factors

Patients with cirrhosis and chronic pain frequently share overlapping comorbidities and risk factors that can independently drive both conditions. Rather than treating pain in isolation, addressing these shared contributors represents a distinct and potentially synergistic therapeutic strategy. Many of these comorbidities are themselves modifiable, and interventions targeting them may improve pain while simultaneously preventing liver disease progression and decompensation.

### Alcohol Use

Heavy alcohol use can contribute to the development of neuropathies or chronic pancreatitis, both of which are significant sources of pain in cirrhosis [113, 114]. Patients may use alcohol to self-manage chronic pain. However, alcohol consumption promotes both acute tolerance, driving escalation of intake, and chronic neurobiological adaptations that perpetuate pain and alcohol craving [115, 116]. Attempts at alcohol abstinence may precipitate hyperalgesia, creating a cycle in which pain reinforces continued use [117]. Collectively, these considerations underscore the importance of integrating addiction medicine alongside liver disease and chronic pain management. Alcohol cessation also has the potential to halt or reverse hepatic fibrosis, particularly in alcohol-associated liver disease, making this one of the few interventions that simultaneously addresses pain risk factors and disease trajectory.

### Metabolic Conditions

The relationship between obesity and chronic pain is complex. Obesity increases biomechanical load on joints and is associated with gut dysbiosis, systemic inflammation, and autonomic dysfunction, all of which may contribute to pain [118] As noted above, exercise-based interventions can be beneficial for both pain and metabolic health in this population [119]. Weight loss through comprehensive strategies, including glucagon-like peptide-1 receptor (GLP-1) agonists, can be considered, though careful monitoring for sarcopenia is warranted in patients with cirrhosis on these agents. For patients with comorbid Type 2 diabetes, glycemic control is important to prevent progression of diabetic neuropathy, a common and undertreated cause of pain in patients with liver disease [120]. In patients with MASLD-related cirrhosis, interventions targeting metabolic risk factors have the added potential to modify disease trajectory.

### Sarcopenia and Frailty

Sarcopenia and chronic pain share mechanisms, including chronic inflammation, inactivity, and central sensitization [121]. Although the relationship has not been studied specifically in cirrhosis, sarcopenia is highly prevalent in this population and contributes to disability and poor quality of life. Comprehensive strategies to improve muscle mass and physical function, including structured exercise and nutritional optimization, may mitigate chronic pain while also addressing frailty, a major driver of morbidity and mortality in advanced liver disease.

### Sleep Disturbances

Sleep disturbances, including insomnia and altered sleep–wake cycles, are common among patients with cirrhosis [122]. There is a well-established bidirectional relationship between sleep and chronic pain in the general population: poor sleep lowers pain thresholds, and pain disrupts sleep [123]. In chronic pain populations with co-occurring insomnia, data suggest that gabapentinoids and CBT for insomnia may be particularly effective [124]. Managing sleep disturbances in patients with cirrhosis requires careful attention to hepatic encephalopathy, which can present with similar symptoms and may confound the clinical picture.

### Depression and Anxiety

Depression and anxiety are prevalent among patients with cirrhosis and are independently associated with pain severity and pain-related disability [1, 125]. The relationship is bidirectional: psychiatric comorbidities amplify the pain experience, and chronic pain worsens mood and anxiety. Strategies that target both pain and psychiatric comorbidities simultaneously, such as CBT and antidepressants, may be more effective than treating either condition in isolation. As noted above, antidepressant selection in patients with cirrhosis requires attention to hepatic metabolism and drug-specific risks.

### Portal Hypertensive Complications

Portal hypertensive complications, including ascites and spontaneous bacterial peritonitis, may themselves be significant sources of pain in patients with cirrhosis. In a large cohort of patients with cirrhosis, abdominal pain was the most common pain location, and those with abdominal pain were more likely to have decompensation [2]. Identifying and treating the underlying cause of pain, including drainage of tense ascites and treatment of infections, is an essential first step before initiating analgesic therapy. Optimization of liver disease management, including portal pressure reduction and prevention of decompensating events, may reduce pain burden while also improving clinical outcomes.

## Best Practices for Pain Management

Incorporating pain management into routine hepatology care presents real barriers including time limitations, competing priorities, variability in expertise, insurance and reimbursement concerns, and patient receptiveness. Yet pain is a central and undertreated concern for many patients with cirrhosis, and effective management can improve quality of life and patient satisfaction. Hepatologists are not typically the primary providers managing chronic pain, but clinicians from other specialties may be less familiar with analgesic safety in the setting of liver dysfunction [126]. The hepatologist therefore plays a critical role in guiding safe, multimodal pain management and coordinating care across specialties.

While structured pain management models have not been studied in hepatology settings, we propose several pragmatic strategies. Clinicians should clarify that acetaminophen 2 g/day and topical analgesics are generally safe in cirrhosis, while reinforcing the risks of NSAIDs, opioids, and gabapentinoids. Patients can also be counseled that addressing shared risk factors and comorbidities—alcohol use, metabolic disease, sleep, and mood—may improve both pain symptoms and liver disease trajectory.

**Fig. 1:**
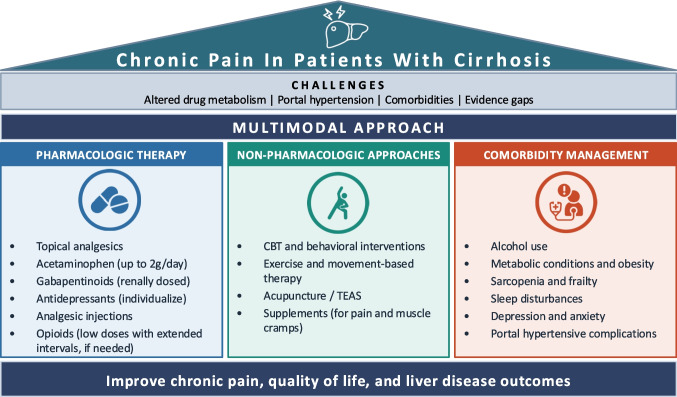
A proposed multimodal approach to chronic pain management in patients with cirrhosis

Within the electronic health record, clinicians should proactively assess and document pain as part of the patient’s problem list, include pain management considerations in clinical notes for other treating providers, and provide standardized after-visit summary instructions outlining multimodal pain management options (Fig. 1).

Given that cirrhosis is a serious, life-limiting illness, early referral to palliative care can help address pain alongside other symptoms.^17^ Where available, integrated care models that embed behavioral health, physical therapy, or pain specialists within hepatology or primary care, may help operationalize multimodal care in this medically complex population. We acknowledge that practice patterns and patient populations vary across settings; therefore, these recommendations should be adapted to the local clinical context.

## Conclusions and Future Directions

Chronic pain is prevalent among patients with cirrhosis, yet the evidence base to guide its management remains remarkably thin. Nearly every pharmacologic class carries theoretical or demonstrated risk in this population, and most prescribing recommendations are based on pharmacokinetic data and expert opinion rather than real-world pharmacoepidemiologic data or clinical trials. Non-pharmacologic modalities, which may be particularly important given these pharmacologic constraints, have been scarcely studied in patients with liver disease. Even the most basic clinical question—which analgesic is safest in patients with cirrhosis—cannot be answered definitively with current data.

Despite these limitations, the available evidence supports an individualized, multimodal approach that integrates pharmacologic management, non-pharmacologic therapies, and treatment of shared comorbidities and risk factors (Fig. 1). No single intervention is likely to be sufficient. Acetaminophen at reduced doses remains the preferred first-line analgesic; when additional pharmacotherapy is needed, selection should be guided by liver disease severity, pain type and comorbidity profile, with close monitoring for adverse effects. Topical therapies and injections warrant consideration where appropriate. Non-pharmacologic approaches, including behavioral interventions, exercise, and acupuncture, should be incorporated early rather than reserved as a last resort. Addressing modifiable comorbidities such as alcohol use, metabolic disease, sleep disturbances, and psychiatric conditions may improve pain while simultaneously altering the trajectory of liver disease.

Several areas warrant prioritization in future research. Both well-designed observational studies leveraging existing clinical data and prospective trials are needed to generate evidence on analgesic safety and efficacy in patients with cirrhosis, as this population continues to be excluded from most drug trials. Equally important is the evaluation of non-pharmacologic interventions, which have demonstrated benefit in other chronic pain populations but remain largely untested in patients with liver disease. Better characterization of pain mechanisms in cirrhosis, including the relative contributions of nociceptive, neuropathic, and nociplastic pain, could help tailor treatment to individual patients rather than relying on a one-size-fits-all approach. The role of supplements and cannabis in this population also requires rigorous evaluation, given their widespread use. Finally, the absence of cirrhosis-specific evidence-based pain management guidelines contributes to significant variability in clinical practice; developing such guidance would be a valuable step toward standardizing care.

Effective pain management in patients with cirrhosis will require not only better evidence but also better integration of care across hepatology, pain medicine, addiction medicine, behavioral health, and palliative care. Clinicians who treat these patients must be willing to move beyond a pharmacotherapy-first approach and embrace the multimodal framework that the complexity of this problem demands.

## Key References

- Barakji JA, Maagaard M, Petersen JJ, et al. Tramadol versus placebo for chronic pain: a systematic review with meta-analysis and trial sequential analysis. BMJ Evid Based Med. 2025;bmjebm-2025–114101.
  - First comprehensive systematic review of tramadol alone for chronic pain, finding only modest pain relief below the minimal clinically important difference with increased serious adverse events.
- Holman A, Parikh ND, Zhao Z, et al. Contemporary management of pain in cirrhosis: Toward precision therapy for pain. Hepatology. 2023;77(1):290–304.
  - Proposes a mechanistic classification framework for pain in cirrhosis (nociceptive, neuropathic, nociplastic) and argues for phenotype-directed treatment approaches.
- Rogal SS, Engel B, Englesbe M, et al. AASLD Practice Guidance on Palliative Care and Symptom-Based Management in Decompensated Cirrhosis. Hepatology. 2024;79(4):960–987.
  - First AASLD guidance addressing symptom management including pain in decompensated cirrhosis.
- Rubin JB, Loeb R, Fenton C, et al. The burden of significant pain in the cirrhosis population: Risk factors, analgesic use, and impact on health care utilization and clinical outcomes. Hepatol Commun. 2024;8(6):e0432.
  - Largest contemporary cohort characterizing pain prevalence, location, and analgesic use in over 5,000 patients with cirrhosis.
- Rubin JB, Loeb R, Keyhani S, Hoggatt KJ, Shen H, Lai JC. Declining Opioid and Rising Nonopioid Analgesic Prescriptions Among 364,000 Veterans with Cirrhosis, 2010–2021. Clin Gastroenterol Hepatol. 2026. 10.1016/j.cgh.2026.03.017.
  - Largest pharmacoepidemiologic study of analgesic prescribing trends in cirrhosis, demonstrating declining opioid and rising nonopioid prescribing over an 11-year period in a national longitudinal cohort.

## Data Availability

No datasets were generated or analysed during the current study.

## References

[1] Rogal SS, Bielefeldt K, Wasan AD, et al. Inflammation, psychiatric symptoms, and opioid use are associated with pain and disability in patients with cirrhosis. Clin Gastroenterol Hepatol. 2015;13(5):1009–16. 25460019 10.1016/j.cgh.2014.10.029 PMC4846465

[2] Rubin JB, Loeb R, Fenton C, et al. The burden of significant pain in the cirrhosis population: risk factors, analgesic use, and impact on health care utilization and clinical outcomes. Hepatol Commun. 2024;8(6):e0432. 38780295 10.1097/HC9.0000000000000432 PMC11124725

[3] Zhang GY, Cortella A, Lai JC, et al. Pain in chronic liver disease compared to other chronic conditions: results from a contemporary nationally representative cohort study. Hepatol Commun. 2025;9(1). 10.1097/HC9.0000000000000605 PMC11637743 39670874

[4] Pu K, Zhu X. Longitudinal analysis of pain symptoms and risk factors in patients with incident liver disease: a nationwide cohort study. Eur J Med Res. 2026. 10.1186/s40001-026-04003-w. 41664217 10.1186/s40001-026-04003-w

[5] Ma J, Björnsson ES, Chalasani N. The safe use of analgesics in patients with cirrhosis: a narrative review. Am J Med. 2024;137(2):99–106. 37918778 10.1016/j.amjmed.2023.10.022

[6] Rogal SS, Beste LA, Youk A, et al. Characteristics of opioid prescriptions to veterans with cirrhosis. Clin Gastroenterol Hepatol. 2019;17(6):1165-74.e3. 30342261 10.1016/j.cgh.2018.10.021 PMC8108399

[7] Rogal SS, Winger D, Bielefeldt K, et al. Pain and opioid use in chronic liver disease. Dig Dis Sci. 2013;58(10):2976–85. 23512406 10.1007/s10620-013-2638-5 PMC3751995

[8] Rubin JB, Lai JC, Shui AM, et al. Cirrhosis inpatients receive more opioids and fewer nonopioid analgesics than patients without cirrhosis. J Clin Gastroenterol. 2023;57(1):48–56. 34653064 10.1097/MCG.0000000000001624 PMC9008074

[9] Rubin JB, Loeb R, Keyhani S, et al. Declining Opioid and Rising Nonopioid Analgesic Prescriptions Among 364,000 Veterans with Cirrhosis, 2010–2021. Clin Gastroenterol Hepatol. 2026.10.1016/j.cgh.2026.03.01741935594

[10] Bosilkovska M, Walder B, Besson M, et al. Analgesics in patients with hepatic impairment: pharmacology and clinical implications. Drugs. 2012;72(12):1645–69. 22867045 10.2165/11635500-000000000-00000

[11] Bergasa NV, Rothman RB, Mukerjee E, et al. Up-regulation of central mu-opioid receptors in a model of hepatic encephalopathy: a potential mechanism for increased sensitivity to morphine in liver failure. Life Sci. 2002;70(14):1701–8. 11991257 10.1016/s0024-3205(02)01487-x

[12] Acharya C, Betrapally NS, Gillevet PM, et al. Chronic opioid use is associated with altered gut microbiota and predicts readmissions in patients with cirrhosis. Aliment Pharmacol Ther. 2017;45(2):319–31. 27868217 10.1111/apt.13858

[13] Moon AM, Jiang Y, Rogal SS, et al. Opioid prescriptions are associated with hepatic encephalopathy in a national cohort of patients with compensated cirrhosis. Aliment Pharmacol Ther. 2020;51(6):652–60. 31960985 10.1111/apt.15639 PMC7047528

[14] Tapper EB, Henderson JB, Parikh ND, et al. Incidence of and risk factors for hepatic encephalopathy in a population-based cohort of Americans with cirrhosis. Hepatol Commun. 2019;3(11):1510–9. 31701074 10.1002/hep4.1425 PMC6824059

[15] Johnson AW, Golzarri Arroyo L, Mahendraker N, et al. Hospital opioid usage and adverse events in patients with end-stage liver disease. J Pain Symptom Manage. 2023;65(4):326-34.e2. 36496114 10.1016/j.jpainsymman.2022.11.026

[16] Haberer JP, Schoeffler P, Couderc E, et al. Fentanyl pharmacokinetics in anaesthetized patients with cirrhosis. Br J Anaesth. 1982;54(12):1267–70. 7171414 10.1093/bja/54.12.1267

[17] Rogal SS, Hansen L, Patel A, et al. AASLD practice guidance: palliative care and symptom-based management in decompensated cirrhosis. Hepatology. 2022;76(3):819–53. 35103995 10.1002/hep.32378 PMC9942270

[18] Fournier JP, Yin H, Nessim SJ, et al. Tramadol for noncancer pain and the risk of hyponatremia. Am J Med. 2015;128(4):418-25.e5. 25460534 10.1016/j.amjmed.2014.10.046

[19] Fournier JP, Azoulay L, Yin H, et al. Tramadol use and the risk of hospitalization for hypoglycemia in patients with noncancer pain. JAMA Intern Med. 2015;175(2):186–93. 25485799 10.1001/jamainternmed.2014.6512

[20] Barakji JA, Maagaard M, Petersen JJ, et al. Tramadol versus placebo for chronic pain: a systematic review with meta-analysis and trial sequential analysis. BMJ Evid Based Med. 2025:bmjebm-2025–114101. 10.1136/bmjebm-2025-114101 41057269

[21] Rubin JB, Lai JC, Shui AM, et al. Patterns of inpatient opioid use and related adverse events among patients with cirrhosis: a propensity-matched analysis. Hepatol Commun. 2021;5(6):1081–94. 34141991 10.1002/hep4.1694 PMC8183179

[22] Mazoit JX, Butscher K, Samii K. Morphine in postoperative patients: pharmacokinetics and pharmacodynamics of metabolites. Anesth Analg. 2007;105(1):70–8. 17578959 10.1213/01.ane.0000265557.73688.32

[23] Petry NM, Bickel WK, Piasecki D, et al. Elevated liver enzyme levels in opioid-dependent patients with hepatitis treated with buprenorphine. Am J Addict. 2000;9(3):265–9. 11000922 10.1080/10550490050148099

[24] Bruce RD, Altice FL. Case series on the safe use of buprenorphine/naloxone in individuals with acute hepatitis C infection and abnormal hepatic liver transaminases. Am J Drug Alcohol Abuse. 2007;33(6):869–74. 17994482 10.1080/00952990701653875

[25] Saxon AJ, Ling W, Hillhouse M, et al. Buprenorphine/Naloxone and methadone effects on laboratory indices of liver health: a randomized trial. Drug Alcohol Depend. 2013;128(1–2):71–6. 22921476 10.1016/j.drugalcdep.2012.08.002 PMC3543467

[26] Rogal SS, Winger D, Bielefeldt K, et al. Healthcare utilization in chronic liver disease: the importance of pain and prescription opioid use. Liver Int. 2013;33(10):1497–503. 23758842 10.1111/liv.12215 PMC3795935

[27] Moon AM, Jiang Y, Rogal SS, et al. In inpatients with cirrhosis opioid use is common and associated with length of stay and persistent use post-discharge. PLoS ONE. 2020;15(2):e0229497. 32101574 10.1371/journal.pone.0229497 PMC7043759

[28] Rubin JB, Aby ES, Barman P, et al. Opioid use and risks in candidates and recipients of liver transplant. Liver Transpl. 2025;31(2):231–41. 38669598 10.1097/LVT.0000000000000388 PMC11518881

[29] Rossi S, Assis DN, Awsare M, et al. Use of over-the-counter analgesics in patients with chronic liver disease: physicians’ recommendations. Drug Saf. 2008;31(3):261–70. 18302450 10.2165/00002018-200831030-00007

[30] Arnman R, Olsson R. Elimination of paracetamol in chronic liver disease. Acta Hepatogastroenterol (Stuttg). 1978;25(4):283–6. 696204

[31] Andreasen PB, Hutters L. Paracetamol (acetaminophen) clearance in patients with cirrhosis of the liver. Acta Med Scand Suppl. 1979;624:99–105. 284720 10.1111/j.0954-6820.1979.tb00728.x

[32] Villeneuve JP, Raymond G, Bruneau J, et al. [Pharmacokinetics and metabolism of acetaminophen in normal, alcoholic and cirrhotic subjects]. Gastroenterol Clin Biol. 1983;7(11):898–902. Pharmacocinétique et métabolisme de l’acétaminophène chez des sujets normaux, alcooliques et cirrhotiques. 6653975

[33] Khalid SK, Lane J, Navarro V, et al. Use of over-the-counter analgesics is not associated with acute decompensation in patients with cirrhosis. Clin Gastroenterol Hepatol. 2009;7(9):994–9. 19394441 10.1016/j.cgh.2009.04.015 PMC3777825

[34] McGill MR, James LP, McCullough SS, et al. Short-term safety of repeated acetaminophen use in patients with compensated cirrhosis. Hepatol Commun. 2022;6(2):361–73. 34558847 10.1002/hep4.1810 PMC8793989

[35] Benson GD. Acetaminophen in chronic liver disease. Clin Pharmacol Ther. 1983;33(1):95–101. 6848304 10.1038/clpt.1983.14

[36] Zimmerman HJ, Maddrey WC. Acetaminophen (paracetamol) hepatotoxicity with regular intake of alcohol: analysis of instances of therapeutic misadventure. Hepatology. 1995;22(3):767–73. 7657281

[37] Kuffner EK, Green JL, Bogdan GM, et al. The effect of acetaminophen (four grams a day for three consecutive days) on hepatic tests in alcoholic patients–a multicenter randomized study. BMC Med. 2007;5:13. 17537264 10.1186/1741-7015-5-13 PMC1894983

[38] Dart RC, Green JL, Kuffner EK, et al. The effects of paracetamol (acetaminophen) on hepatic tests in patients who chronically abuse alcohol - a randomized study. Aliment Pharmacol Ther. 2010;32(3):478–86. 20491750 10.1111/j.1365-2036.2010.04364.x

[39] Fan XF, Xu SK, Fu SA, et al. Early acetaminophen administration and mortality outcomes in critically ill patients with cirrhosis: a retrospective analysis from the MIMIC-IV database. BMC Gastroenterol. 2025;25(1):522. 40670916 10.1186/s12876-025-04113-5 PMC12265238

[40] Park GC, Chung JW, Jang ES, et al. Association between adverse outcomes of hepatitis A and acetaminophen use: A population-based cohort study. Dig Liver Dis. 2023;55(10):1368–74. 37088594 10.1016/j.dld.2023.03.017

[41] Lee WM, Barnard C, Rule JA, et al. Association of Acetaminophen (Paracetamol) Use With Severity and Outcomes in Patients With Viral Hepatitis-Associated Acute Liver Failure. Am J Gastroenterol. 2025;120(3):584–92. 38994834 10.14309/ajg.0000000000002941 PMC11724933

[42] Antillon M, Cominelli F, Reynolds TB, et al. Comparative acute effects of diflunisal and indomethacin on renal function in patients with cirrhosis and ascites. Am J Gastroenterol. 1989;84(2):153–5. 2916525

[43] Elia C, Graupera I, Barreto R, et al. Severe acute kidney injury associated with non-steroidal anti-inflammatory drugs in cirrhosis: A case-control study. J Hepatol. 2015;63(3):593–600. 25872166 10.1016/j.jhep.2015.04.004

[44] Ackerman Z, Cominelli F, Reynolds TB. Effect of misoprostol on ibuprofen-induced renal dysfunction in patients with decompensated cirrhosis: results of a double-blind placebo-controlled parallel group study. Am J Gastroenterol. 2002;97(8):2033–9. 12190173 10.1111/j.1572-0241.2002.05847.x

[45] Lieber SR, Jiang Y, Moon A, et al. Antiplatelet medications are associated with bleeding and decompensation events among patients with cirrhosis. J Clin Gastroenterol. 2022;56(7):627–34. 34049373 10.1097/MCG.0000000000001558 PMC8627524

[46] De Lédinghen V, Heresbach D, Fourdan O, et al. Anti-inflammatory drugs and variceal bleeding: a case-control study. Gut. 1999;44(2):270–3. 9895389 10.1136/gut.44.2.270 PMC1727398

[47] Lee YC, Chang CH, Lin JW, et al. Non-steroidal anti-inflammatory drugs use and risk of upper gastrointestinal adverse events in cirrhotic patients. Liver Int. 2012;32(5):859–66. 22226322 10.1111/j.1478-3231.2011.02739.x

[48] Bockbrader HN, Wesche D, Miller R, et al. A comparison of the pharmacokinetics and pharmacodynamics of pregabalin and gabapentin. Clin Pharmacokinet. 2010;49(10):661–9. 20818832 10.2165/11536200-000000000-00000

[49] Holman A, Parikh N, Clauw DJ, et al. Contemporary management of pain in cirrhosis: toward precision therapy for pain. Hepatology. 2023;77(1):290–304. 35665522 10.1002/hep.32598 PMC9970025

[50] Strahan AE, Rikard SM, Schmit K, et al. Trends in dispensed gabapentin prescriptions in the United States, 2010 to 2024. Ann Intern Med. 2025;178(12):1816–8. 41021923 10.7326/ANNALS-25-01750

[51] Wiffen PJ, Derry S, Bell RF, et al. Gabapentin for chronic neuropathic pain in adults. Cochrane Database Syst Rev. 2017;6(6):Cd007938. 28597471 10.1002/14651858.CD007938.pub4 PMC6452908

[52] American Geriatrics Society. 2023 updated AGS Beers Criteria® for potentially inappropriate medication use in older adults. J Am Geriatr Soc. 2023;71(7):2052–81. 37139824 10.1111/jgs.18372 PMC12478568

[53] Ahn S, Hong YH, Lee DH, et al. Efficacy and safety of Pregabalin for muscle cramps in liver cirrhosis: a double-blind randomized controlled trial. J Korean Med Sci. 2022;37(7):e56. 35191232 10.3346/jkms.2022.37.e56 PMC8860769

[54] Mundada K, Goel A, Paliwal VK, et al. Short course of low-dose pregabalin is effective for the treatment of restless leg syndrome in patients with cirrhosis: a pilot study. J Gastroenterol Hepatol. 2022;37(5):933–7. 35174537 10.1111/jgh.15803

[55] Chen JY, Ren Y, Yan P, et al. Tricyclic antidepressant use and the risk of fibrosis progression in Hepatitis C-infected persons: results from ERCHIVES. J Viral Hepat. 2018;25(7):825–33. 29478294 10.1111/jvh.12884 PMC6019114

[56] Rao AK, Rockey D. Tricyclic antidepressants (TCAs) are associated with reduced portal hypertension complications and mortality in patients with cirrhosis. Am J Med Sci. 2026;371:S354–5.

[57] Suri A, Reddy S, Gonzales C, et al. Duloxetine pharmacokinetics in cirrhotics compared with healthy subjects. Int J Clin Pharmacol Ther. 2005;43(2):78–84. 15726876 10.5414/cpp43078

[58] Vuppalanchi R, Hayashi PH, Chalasani N, et al. Duloxetine hepatotoxicity: a case-series from the Drug-Induced Liver Injury Network. Aliment Pharmacol Ther. 2010;32(9):1174–83. 20815829 10.1111/j.1365-2036.2010.04449.x PMC3773985

[59] Xue F, Strombom I, Turnbull B, et al. Duloxetine for depression and the incidence of hepatic events in adults. J Clin Psychopharmacol. 2011;31(4):517–22. 21694615 10.1097/JCP.0b013e31822347d9

[60] Jiang HY, Chen HZ, Hu XJ, et al. Use of selective serotonin reuptake inhibitors and risk of upper gastrointestinal bleeding: a systematic review and meta-analysis. Clin Gastroenterol Hepatol. 2015;13(1):42-50.e3. 24993365 10.1016/j.cgh.2014.06.021

[61] Saarto T, Wiffen PJ. Antidepressants for neuropathic pain. Cochrane Database Syst Rev. 2007;2007(4):Cd005454. 17943857 10.1002/14651858.CD005454.pub2 PMC10576544

[62] Campbell BJ, Rowbotham M, Davies PS, et al. Systemic absorption of topical lidocaine in normal volunteers, patients with post-herpetic neuralgia, and patients with acute herpes zoster. J Pharm Sci. 2002;91(5):1343–50. 11977110 10.1002/jps.10133

[63] Derry S, Wiffen PJ, Kalso EA, et al. Topical analgesics for acute and chronic pain in adults - an overview of Cochrane Reviews. Cochrane Database Syst Rev. 2017;5(5):Cd008609. 10.1002/14651858.CD008609.pub2 PMC6481750 28497473

[64] Rannou F, Pelletier JP, Martel-Pelletier J. Efficacy and safety of topical NSAIDs in the management of osteoarthritis: evidence from real-life setting trials and surveys. Semin Arthritis Rheum. 2016;45(4 Suppl):S18-21. 26806189 10.1016/j.semarthrit.2015.11.007

[65] Ferraro MC, Cashin AG, Visser EJ, et al. Ketamine and other NMDA receptor antagonists for chronic pain. Cochrane Database Syst Rev. 2025;8(8):Cd015373. 40819842 10.1002/14651858.CD015373.pub2 PMC12358209

[66] Peskin E, Gudin J, Schatman ME. Increased demand for ketamine infusions and associated complexities. J Pain Res. 2023;16:295–9. 36744115 10.2147/JPR.S403323 PMC9891072

[67] Noppers IM, Niesters M, Aarts LP, et al. Drug-induced liver injury following a repeated course of ketamine treatment for chronic pain in CRPS type 1 patients: a report of 3 cases. Pain. 2011;152(9):2173–8. 21546160 10.1016/j.pain.2011.03.026

[68] Wong GL, Tam YH, Ng CF, et al. Liver injury is common among chronic abusers of ketamine. Clin Gastroenterol Hepatol. 2014;12(10):1759-62.e1. 24534547 10.1016/j.cgh.2014.01.041

[69] Teymouri A, Nasoori H, Fakheri M, et al. Features of biliary tract diseases in ketamine abusers: a systematic review of case reports. J Med Case Rep. 2024;18(1):84. 38431685 10.1186/s13256-024-04421-6 PMC10909254

[70] Cohen SP, Vase L, Hooten WM. Chronic pain: an update on burden, best practices, and new advances. Lancet. 2021;397(10289):2082–97. 34062143 10.1016/S0140-6736(21)00393-7

[71] Yui JC, Preskill C, Greenlund LS. Arthrocentesis and joint injection in patients receiving direct oral anticoagulants. Mayo Clin Proc. 2017;92(8):1223–6. 28778256 10.1016/j.mayocp.2017.04.007

[72] Micu A, Micu MC, Bodozs G, et al. To stop or not to stop novel oral anticoagulants prior to performing joint interventional maneuvers? Evidence from a prospective study that the therapy can be maintained. Clin Rheumatol. 2024;43(9):2983–92. 39008221 10.1007/s10067-024-07048-6

[73] Tsui BC, Kirkham K, Kwofie MK, et al. Practice advisory on the bleeding risks for peripheral nerve and interfascial plane blockade: evidence review and expert consensus. Can J Anaesth. 2019;66(11):1356–84. 31452012 10.1007/s12630-019-01466-w

[74] Goodman BS, House LM, Vallabhaneni S, et al. Anticoagulant and antiplatelet management for spinal procedures: a prospective, descriptive study and interpretation of guidelines. Pain Med. 2017;18(7):1218–24. 28339551 10.1093/pm/pnw227

[75] Gandy M, Pang STY, Scott AJ, et al. Internet-delivered cognitive and behavioural based interventions for adults with chronic pain: a systematic review and meta-analysis of randomized controlled trials. Pain. 2022;163(10):e1041–53. 35121696 10.1097/j.pain.0000000000002606

[76] DeBar LL, Mayhew M, Wellman RD, et al. Telehealth and online cognitive behavioral therapy-based treatments for high-impact chronic pain: a randomized clinical trial. JAMA. 2025;334(7):592–605. 40699570 10.1001/jama.2025.11178 PMC12287937

[77] Burgess DJ, Calvert C, Hagel Campbell EM, et al. Telehealth mindfulness-based interventions for chronic pain: the lamp randomized clinical trial. JAMA Intern Med. 2024;184(10):1163–73. 39158851 10.1001/jamainternmed.2024.3940 PMC11334014

[78] Zhu M, Wong SY, Zhong CC, et al. Which type and dosage of mindfulness-based interventions are most effective for chronic pain? A systematic review and network meta-analysis. J Psychosom Res. 2025;191:112061. 40010103 10.1016/j.jpsychores.2025.112061

[79] Rogal SS, Chinman MJ, DeMonte W, et al. Using intervention mapping to develop a novel pain self-management intervention for people with cirrhosis. Dig Dis Sci. 2022;67(11):5063–78.35147816 10.1007/s10620-022-07380-4

[80] Aamann L, Dam G, Borre M, et al. Resistance training increases muscle strength and muscle size in patients with liver cirrhosis. Clin Gastroenterol Hepatol. 2020;18(5):1179-87. e6. 31394282 10.1016/j.cgh.2019.07.058

[81] Chen HW, Ferrando A, White MG, et al. Home-based physical activity and diet intervention to improve physical function in advanced liver disease: a randomized pilot trial. Dig Dis Sci. 2020;65(11):3350–9. 31907774 10.1007/s10620-019-06034-2

[82] Balaraja S, Taneja S, Anand A, et al. Yoga is non inferior to exercise in improving hepatic steatosis in MASLD-An open label, non-inferiority, randomised controlled trial. J Clin Exp Hepatol. 2025;15.

[83] Bayat Z, Mahdioun SS, Nemati H, et al. Effects of cyclic yoga on selected metabolic and hepatic parameters in diabetic women with fatty liver diseases: a clinical trial. J Clin Transl Endocrinol. 2026. 10.1016/j.jcte.2026.100431. 41658040 10.1016/j.jcte.2026.100431 PMC12874436

[84] Tankha H, Gaskins D, Shallcross A, et al. Effectiveness of virtual yoga for chronic low back pain: a randomized clinical trial. JAMA Netw Open. 2024;7(11):e2442339. 39485352 10.1001/jamanetworkopen.2024.42339 PMC11530940

[85] Tilbrook HE, Cox H, Hewitt CE, et al. Yoga for chronic low back pain: a randomized trial. Ann Intern Med. 2011;155(9):569–78. 22041945 10.7326/0003-4819-155-9-201111010-00003

[86] Haake M, Müller H-H, Schade-Brittinger C. German acupuncture trials (GERAC) for chronic low back pain: randomized, multicenter, blinded, parallel-group trial with 3 groups. Arch Intern Med. 2007;167(17):1892–8. 17893311 10.1001/archinte.167.17.1892

[87] Brinkhaus B, Witt CM, Jena S, et al. Acupuncture in patients with chronic low back pain: a randomized controlled trial. Arch Intern Med. 2006;166(4):450–7. 16505266 10.1001/archinte.166.4.450

[88] Hsieh HT, Chou HJ, Wu PY, et al. Bleeding risk after acupuncture in patients taking anticoagulant drugs: a case control study based on real-world data. Complement Ther Med. 2023;74:102951. 37141924 10.1016/j.ctim.2023.102951

[89] Zhu L, Li J, Wang ZQ, et al. Treatment of moderate-to-severe pain in hepatocellular carcinoma with transcutaneous electrical acupoint stimulation: a randomized controlled trial. J Pain Res. 2024;17:1583–94. 38707266 10.2147/JPR.S456874 PMC11067922

[90] Xie L, Wang X, Chu J, et al. Effects of omega-3 fatty acids on chronic pain: a systematic review and meta-analysis. Front Med. 2025;12:1654661. 10.3389/fmed.2025.1654661 PMC12627051 41267881

[91] Goldberg RJ, Katz J. A meta-analysis of the analgesic effects of omega-3 polyunsaturated fatty acid supplementation for inflammatory joint pain. Pain. 2007;129(1–2):210–23. 17335973 10.1016/j.pain.2007.01.020

[92] Feng J, Li Z, Tian L, et al. Efficacy and safety of curcuminoids alone in alleviating pain and dysfunction for knee osteoarthritis: a systematic review and meta-analysis of randomized controlled trials. BMC Complement Med Ther. 2022;22(1):276. 10.1186/s12906-022-03740-9 PMC9580113 36261810

[93] Kuptniratsaikul V, Dajpratham P, Taechaarpornkul W, et al. Efficacy and safety of *Curcuma domestica* extracts compared with ibuprofen in patients with knee osteoarthritis: a multicenter study. Clin Interv Aging. 2014;9:451–8. 24672232 10.2147/CIA.S58535 PMC3964021

[94] Yong WC, Sanguankeo A, Upala S. Effect of vitamin D supplementation in chronic widespread pain: a systematic review and meta-analysis. Clin Rheumatol. 2017;36(12):2825–33. 28812209 10.1007/s10067-017-3754-y

[95] Li S, Li Q, Li Y, et al. Acetyl-L-carnitine in the treatment of peripheral neuropathic pain: a systematic review and meta-analysis of randomized controlled trials. PLoS ONE. 2015;10(3):e0119479. 25751285 10.1371/journal.pone.0119479 PMC4353712

[96] Miller KC, Mack GW, Knight KL, et al. Reflex inhibition of electrically induced muscle cramps in hypohydrated humans. Med Sci Sports Exerc. 2010;42(5):953–61. 19997012 10.1249/MSS.0b013e3181c0647e

[97] McKenney MA, Miller KC, Deal JE, et al. Plasma and electrolyte changes in exercising humans after ingestion of multiple boluses of pickle juice. J Athl Train. 2015;50(2):141–6. 25562454 10.4085/1062-6050-50.2.07 PMC4495441

[98] Tapper EB, Salim N, Baki J, et al. Pickle juice intervention for cirrhotic cramps reduction: The PICCLES randomized controlled trial. Am J Gastroenterol. 2022;117(6):895–901. 35416793 10.14309/ajg.0000000000001781 PMC11214544

[99] De Luca A, Pierno S, Camerino DC. Effect of taurine depletion on excitation-contraction coupling and C1− conductance of rat skeletal muscle. Eur J Pharmacol. 1996;296(2):215–22. 8838459 10.1016/0014-2999(95)00702-4

[100] Vidot H, Cvejic E, Carey S, et al. Randomised clinical trial: oral taurine supplementation versus placebo reduces muscle cramps in patients with chronic liver disease. Aliment Pharmacol Ther. 2018;48(7):704–12. 30136291 10.1111/apt.14950

[101] Hidaka H, Nakazawa T, Kutsukake S, et al. The efficacy of nocturnal administration of branched-chain amino acid granules to improve quality of life in patients with cirrhosis. J Gastroenterol. 2013;48(2):269–76. 22825550 10.1007/s00535-012-0632-x

[102] Gluud LL, Dam G, Les I, et al. Branched‐chain amino acids for people with hepatic encephalopathy. Cochrane Database Syst Rev. 2015(9). 10.1002/14651858.CD001939.pub3 26377410

[103] Aamann L, Deshpande N, Dam G, et al. Branched-chain amino acids for people with cirrhosis and hepatic encephalopathy. Cochrane Database Syst Rev. 2026;1(1):Cd001939. 41542879 10.1002/14651858.CD001939.pub5 PMC12809873

[104] Sivandzadeh GR, Shahsavari A, Meftah E, et al. Effect of L-carnitine supplementation on muscle cramps in liver cirrhosis: results from a retrospective cohort study. BMC Gastroenterol. 2025;25(1):150. 40059171 10.1186/s12876-025-03730-4 PMC11890553

[105] Nakanishi H, Kurosaki M, Tsuchiya K, et al. L-carnitine reduces muscle cramps in patients with cirrhosis. Clin Gastroenterol Hepatol. 2015;13(8):1540–3. 25496816 10.1016/j.cgh.2014.12.005

[106] Wang L, Hong PJ, May C, et al. Medical cannabis or cannabinoids for chronic non-cancer and cancer related pain: a systematic review and meta-analysis of randomised clinical trials. BMJ. 2021;374:n1034. 34497047 10.1136/bmj.n1034

[107] Adejumo AC, Adegbala OM, Adejumo KL, et al. Reduced incidence and better liver disease outcomes among chronic HCV infected patients who consume cannabis. Can J Gastroenterol Hepatol. 2018;2018:9430953. 30345261 10.1155/2018/9430953 PMC6174743

[108] Adejumo AC, Ajayi TO, Adegbala OM, et al. Cannabis use is associated with reduced prevalence of progressive stages of alcoholic liver disease. Liver Int. 2018;38(8):1475–86. 29341392 10.1111/liv.13696

[109] Adejumo AC, Alliu S, Ajayi TO, et al. Cannabis use is associated with reduced prevalence of non-alcoholic fatty liver disease: a cross-sectional study. PLoS One. 2017;12(4):e0176416. 28441459 10.1371/journal.pone.0176416 PMC5404771

[110] Sobotka LA, Mumtaz K, Hinton A, et al. Cannabis use may reduce healthcare utilization and improve hospital outcomes in patients with cirrhosis. Ann Hepatol. 2021;23:100280. 33157269 10.1016/j.aohep.2020.10.008

[111] Paladiya R, Singh A, Changela M, et al. Cannabis use in metabolic dysfunction-associated steatotic liver disease: friend or foe? A retrospective analysis. J Clin Gastroenterol. 2025. 10.1097/MCG.0000000000002321 41453014

[112] Choi CJ, Weiss SH, Nasir UM, et al. Cannabis use history is associated with increased prevalence of ascites among patients with nonalcoholic fatty liver disease: a nationwide analysis. World J Hepatol. 2020;12(11):993–1003. 33312424 10.4254/wjh.v12.i11.993 PMC7701971

[113] Yamamiya A, Tominaga K, Hoshi K, et al. The risk factors for progression to chronic pancreatitis in patients with past-history of acute pancreatitis: a retrospective analysis based on mechanistic definition. J Clin Med. 2022. 10.3390/jcm11082209. 35456301 10.3390/jcm11082209 PMC9032682

[114] Chopra K, Tiwari V. Alcoholic neuropathy: possible mechanisms and future treatment possibilities. Br J Clin Pharmacol. 2012;73(3):348–62. 21988193 10.1111/j.1365-2125.2011.04111.x PMC3370340

[115] Williams MK, Vitus D, Ferguson E, et al. Acute tolerance to the analgesic effects of alcohol. J Stud Alcohol Drugs. 2021;82(3):422–30. 34100711 10.15288/jsad.2021.82.422 PMC8328235

[116] De Aquino JP, Sloan ME, Nunes JC, et al. Alcohol use disorder and chronic pain: an overlooked epidemic. Am J Psychiatry. 2024;181(5):391–402. 38706339 10.1176/appi.ajp.20230886 PMC11521207

[117] Jochum T, Boettger MK, Burkhardt C, et al. Increased pain sensitivity in alcohol withdrawal syndrome. Eur J Pain. 2010;14(7):713–8. 20018536 10.1016/j.ejpain.2009.11.008

[118] Malfliet A, Quiroz Marnef A, Nijs J, et al. Obesity hurts: the why and how of integrating weight reduction with chronic pain management. Phys Ther. 2021. 10.1093/ptj/pzab198. 34403478 10.1093/ptj/pzab198

[119] Paley CA, Johnson MI. Physical activity to reduce systemic inflammation associated with chronic pain and obesity: a narrative review. Clin J Pain. 2016;32(4):365–70. 25988939 10.1097/AJP.0000000000000258

[120] Carvajal-Moreno L, Coheña-Jiménez M, García-Ventura I, et al. Prevention of peripheral distal polyneuropathy in patients with diabetes: a systematic review. J Clin Med. 2022. 10.3390/jcm11061723. 35330052 10.3390/jcm11061723 PMC8948704

[121] Chen J, Wang X, Xu Z. Sarcopenia and chronic pain in the elderly: a systematic review and meta-analysis. J Pain Res. 2023;16:3569–81. 37908777 10.2147/JPR.S435866 PMC10614663

[122] Ghabril M, Jackson M, Gotur R, et al. Most individuals with advanced cirrhosis have sleep disturbances, which are associated with poor quality of life. Clin Gastroenterol Hepatol. 2017;15(8):1271-8.e6. 28167158 10.1016/j.cgh.2017.01.027 PMC5872836

[123] Runge N, Ahmed I, Saueressig T, et al. The bidirectional relationship between sleep problems and chronic musculoskeletal pain: a systematic review with meta-analysis. Pain. 2024;165(11):2455–67. 38809241 10.1097/j.pain.0000000000003279

[124] Husak AJ, Bair MJ. Chronic pain and sleep disturbances: a pragmatic review of their relationships, comorbidities, and treatments. Pain Med. 2020;21(6):1142–52. 31909797 10.1093/pm/pnz343

[125] Hernaez R, Kramer JR, Khan A, et al. Depression and anxiety are common among patients with cirrhosis. Clin Gastroenterol Hepatol. 2022;20(1):194-203. e1. 32835845 10.1016/j.cgh.2020.08.045 PMC8210475

[126] Thomson MJ, Lok ASF, Tapper EB. Appropriate and potentially inappropriate medication use in decompensated cirrhosis. Hepatology. 2021;73(6):2429–40. 32911564 10.1002/hep.31548 PMC7943648

